# PX-12-induced HeLa cell death is associated with oxidative stress and GSH depletion

**DOI:** 10.3892/ol.2013.1637

**Published:** 2013-10-21

**Authors:** HYE RIM SHIN, BO RA YOU, WOO HYUN PARK

**Affiliations:** Department of Physiology, Medical School, Research Institute for Endocrine Sciences, Chonbuk National University, Jeonju 561-180, Republic of Korea

**Keywords:** PX-12, reactive oxygen species, thioredoxin, cell death, HeLa

## Abstract

PX-12, as an inhibitor of thioredoxin (Trx), has antitumor activity. However, little is known about the toxicological effect of PX-12 on cervical cancer cells. In the present study, the growth inhibitory effects of PX-12 on HeLa cervical cancer cells in association with reactive oxygen species (ROS) and glutathione (GSH) levels were investigated. Based on MTT assays, PX-12 inhibited the growth of HeLa cells with an IC_50_ value of ~7 μM at 72 h. DNA flow cytometry analysis indicated that 5 and 10 μM PX-12 significantly induced a G2/M phase arrest of the cell cycle. PX-12 also increased the number of dead cells and annexin V-fluorescein isothiocyanate-positive cells, which was accompanied by the loss of mitochondrial membrane potential. All the investigated caspase inhibitors significantly rescued certain cells from PX-12-induced HeLa cell death. With respect to ROS and GSH levels, PX-12 increased ROS levels (including O_2_^•−^) in HeLa cells and induced GSH depletion. N-acetyl cysteine markedly reduced the levels of O_2_^•−^ in PX-12-treated HeLa cells, and prevented apoptotic cell death and GSH depletion in these cells. By contrast, L-buthionine sulfoximine intensified cell death and GSH depletion in PX-12-treated HeLa cells. To conclude, this is the first study to demonstrate that PX-12 inhibits the growth of HeLa cells via G2/M phase arrest, as well as inhibiting apoptosis; the effect was associated with intracellular increases in ROS levels and GSH depletion.

## Introduction

Reactive oxygen species (ROS) are highly reactive oxygen free radicals or non-radical molecules, which include hydrogen peroxide (H_2_O_2_), superoxide anion (O_2_^•−^) and hydroxyl radical (^•^OH) (1). These molecules regulate a number of cellular events, including transcription factor activation, gene expression, differentiation and cell proliferation (2,3). ROS are mainly formed as by-products of the respiratory chain during oxidative phosphorylation in the form of O_2_^•−^ or are specifically produced by oxidases, such as nicotine adenine diphosphate (NADPH) oxidase, xanthine oxidase and arachidonic acid oxygenases (4). Excessive ROS production induces cellular damage and death (5,6). Therefore, there are various antioxidants and systems to control excessive ROS levels.

Thioredoxin (Trx) is a low molecular weight (10- to 12-kDa) redox protein (7), which affects cell growth and proliferation by regulating the redox status in cells (8). Trx has two main isoforms: The cytosolic form, Trx-1, and the mitochondrial form, Trx-2 (9). These Trxs are reduced back by Trx reductase and NADPH following the reduction of oxidative target proteins (10,11). It has been reported that Trx-1 is implicated in cell survival, tumor development, angiogenesis and chemoresistance (12,13). Numerous studies have demonstrated that the overexpression of Trx occurs in a variety of cancer types, including gastric and lung cancers (8,14). PX-12 (1-methylpropyl 2-imidazolyl disulfide) is an irreversible Trx-1 inhibitor, which has antitumor properties (15). PX-12 decreased the activity of Trx-1 by thioalkylating the critical cysteine residue (Cys73) in this protein or by increasing the dimerization of its oxidative form. It has also been reported that PX-12 decreases hypoxia-inducible factor-1α transactivation and vascular endothelial growth factor (16,17). Therefore, PX-12 has been clinically tested in colorectal, lung and pancreatic cancers (18,19).

Cervical cancer is a major cause of mortality in females worldwide. Its carcinogenesis is associated with excessive inflammation mediated by ROS. An increase in Trx-1 levels has been observed in cervical cancer patients compared with a control group (20). However, little is known about the cellular effect of PX-12 in cervical cancer. PX-12-induced cell death in cervical cancer cells may be toxicologically attractive in relation to intracellular ROS levels. Therefore, in the present study, the effects of PX-12 on cell growth and death were investigated in human cervical adenocarcinoma HeLa cells. The effects of various caspase inhibitors (pan-caspase and caspase-3, -8 and -9), N-acetyl cysteine (NAC; a well known antioxidant) and L-buthionine sulfoximine [BSO; an inhibitor of glutathione (GSH) synthesis] were also evaluated in PX-12-treated HeLa cells with respect to cell growth, cell death and ROS and GSH levels.

## Materials and methods

### Cell culture

Human cervical adenocarcinoma HeLa cells were obtained from the American Type Culture Collection (Manassas, VA, USA) and maintained in a humidified incubator containing 5% CO_2_ at 37°C. The HeLa cells were cultured in RPMI-1640 (Sigma-Aldrich, St. Louis, MO, USA) supplemented with 10% fetal bovine serum (Sigma-Aldrich) and 1% penicillin-streptomycin (Gibco BRL, Grand Island, NY, USA). The cells were routinely grown in 100-mm plastic tissue culture dishes (Nunc, Roskilde, Denmark) and harvested with a solution of trypsin-EDTA while in a logarithmic phase of growth.

### Reagents

PX-12 was purchased from Tocris Bioscience (Bristol, UK) and was dissolved in dimethyl sulfoxide (DMSO; Sigma-Aldrich) at 100 mM as a stock solution. The pan-caspase inhibitor benzyloxycarbonyl-Val-Ala-Asp-fluoromethylketone (Z-VAD-FMK), caspase-3 inhibitor benzyloxycarbonyl-Asp-Glu-Val-Asp-fluoromethylketone (Z-DEVD-FMK), caspase-8 inhibitor benzyloxycarbonyl-Ile-Glu-Thr-Asp-fluoromethylketone (Z-IETD-FMK) and caspase-9 inhibitor benzyloxycarbonyl-Leu-Glu-His-Asp-fluoromethylketone (Z-LEHD-FMK) were obtained from R&D Systems Inc. (Minneapolis, MN, USA) and were dissolved in DMSO at 10 mM to serve as stock solutions. NAC and BSO were obtained from Sigma-Aldrich. NAC was dissolved in 20 mM HEPES buffer (pH 7.0) and BSO was dissolved in water. Based on previous studies (21,22), cells were pretreated with 15 μM caspase inhibitors, 2 mM NAC or 10 μM BSO for 1 h prior to treatment with PX-12. DMSO (0.2%) was used as a control vehicle and it did not affect cell growth or death.

### Growth inhibition assay

The effect of PX-12 on cell growth was determined by measuring 3-(4,5-dimethylthiazol-2-yl)-2,5-diphenyltetrazolium bromide (MTT; Sigma-Aldrich) absorbance in living cells as described previously (23). In brief, 1×10^4^ cells/well were seeded in 96-well microtiter plates (Nunc). Following exposure to the designated doses of PX-12 for the indicated times, MTT solution [20 μl: 2 mg/ml in phosphate-buffered saline (PBS)] was added to each well. The plates were incubated for 3 h at 37°C. Medium was withdrawn from the plates by pipetting and 200 μl DMSO was added to each well to solubilize the formazan crystals. The optical density was measured at 570 nm using a microplate reader (Synergy™ 2, BioTek Instruments Inc., Winooski, VT, USA). The cell population was visualized under a light microscope at ×400 magnification (FLoid^®^ Cell Imaging Station, Life Technologies Corporation, Carlsbad, CA, USA).

### Cell cycle and sub-G1 cell analysis

Cell cycle and sub-G1 cell analysis were determined by propidium iodide (PI, Ex/Em=488/617 nm; Sigma-Aldrich) staining as described previously (24). In brief, 1×10^6^ cells in a 60-mm culture dish (Nunc) were incubated with the designated doses of PX-12 for 72 h. Total cells, including floating cells, were then washed with PBS and fixed in 70% (v/v) ethanol. Cells were washed again with PBS, then incubated with PI (10 μg/ml) with simultaneous RNase treatment at 37°C for 30 min. Cellular DNA content was measured using a FACStar flow cytometer (Becton Dickinson, Franklin Lakes, NJ, USA) and analyzed using Lysis II and CellFit software (Becton Dickinson).

### Annexin V-fluorescein isothiocyanate (FITC)/PI staining for the detection of cell death

Apoptotic cell death was determined by staining cells with annexin V-FITC (Ex/Em=488/519 nm; Invitrogen Life Technologies, Camarillo, CA, USA) as described previously (25). In brief, 1×10^6^ cells in a 60-mm culture dish were incubated with the designated doses of PX-12 for 72 h with or without 15 μM each caspase inhibitor, 2 mM NAC or 10 μM BSO. Cells were washed twice with cold PBS and then resuspended in 500 μl binding buffer [10 mM HEPES/NaOH (pH 7.4), 140 mM NaCl and 2.5 mM CaCl_2_] at a concentration of 1×10^6^ cells/ml. Annexin V-FITC (5 μl) and PI (1 μg/ml) were then added and the cells were analyzed with the FACStar flow cytometer. Viable cells were negative for PI and annexin V, apoptotic cells were positive for annexin V and negative for PI, whereas late apoptotic dead cells exhibited high annexin V and PI labeling. Non-viable cells that underwent necrosis, were positive for PI and negative for annexin V.

### Measurement of the mitochondrial membrane potential (MMP)

MMP was measured by a rhodamine 123 fluorescent dye (Ex/Em=485/535 nm; Sigma-Aldrich) as described previously (25,26). In brief, 1×10^6^ cells in a 60-mm culture dish were incubated with the designated doses of PX-12 for 72 h with or without 15 μM each caspase inhibitor, 2 mM NAC or 10 μM BSO. Cells were washed twice with PBS and incubated with rhodamine 123 (0.1 μg/ml) at 37°C for 30 min. Rhodamine 123 staining intensity was determined using the FACStar flow cytometer. The cells that were rhodamine 123-negative were indicated to have lost MMP.

### Detection of intracellular ROS levels

Intracellular ROS levels were detected using an oxidation-sensitive fluorescent probe dye, 2′,7′-dichlorodihydrofluorescein diacetate (H_2_DCFDA; Ex/Em=495/529 nm; Invitrogen Life Technologies) and dihydroethidium (DHE; Ex/Em=518/605 nm; Invitrogen Life Technologies) as previously described (25,27). DHE is highly selective for O_2_^•−^ among ROS. In brief, 1×10^6^ cells in a 60-mm culture dish were incubated with the designated doses of PX-12 for 72 h with or without 15 μM each caspase inhibitor, 2 mM NAC or 10 μM BSO. Cells were then washed in PBS and incubated with 20 μM H_2_DCFDA or DHE at 37°C for 30 min. H_2_DCFDA or DHE fluorescence was assessed using the FACStar flow cytometer. ROS and O_2_^•−^ levels were expressed as mean fluorescence intensity, which was calculated by CellQuest software (Becton Dickinson).

### Detection of intracellular GSH

Intracellular GSH levels were analyzed using a 5-chloromethylfluorescein diacetate (CMFDA) dye (Ex/Em=522/595 nm; Invitrogen Life Technologies) as previously described (27,28). In brief, 1×10^6^ cells in a 60-mm culture dish were incubated with the designated doses of PX-12 for 72 h with or without 15 μM each caspase inhibitor, 2 mM NAC or 10 μM BSO. Cells were then washed with PBS and incubated with 5 μM CMFDA at 37°C for 30 min. CMFDA fluorescence intensity was determined using the FACStar flow cytometer. Negative CMFDA staining (GSH-depletion) of cells was expressed as the percentage of CMFDA-negative cells.

### Statistical analysis

Results represent the mean of at least three independent experiments (mean ± standard deviation). Data were analyzed using Instat software (GraphPad Prism 4, San Diego, CA, USA). Student’s t-test or one-way analysis of variance with post hoc analysis using Tukey’s multiple comparison test were used for parametric data. P<0.05 was considered to indicate a statistically significant difference.

## Results

### Effects of PX-12 on cell growth and cell cycle distribution in HeLa cells

**Figure 1:**
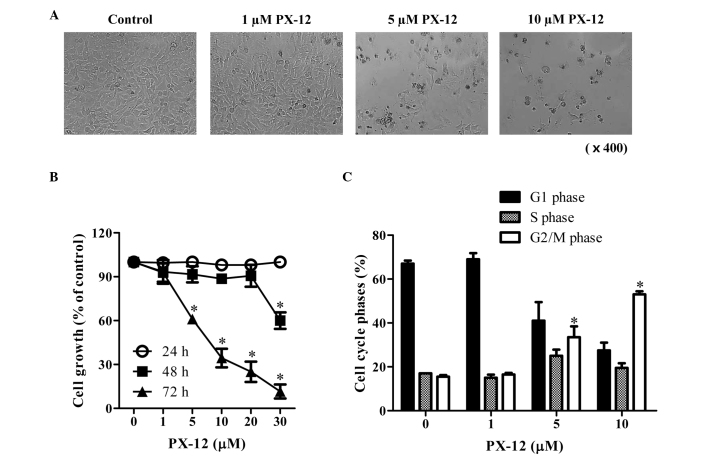
Effects of PX-12 on cell growth and cell cycle distribution in HeLa cells. Exponentially growing cells were treated with the indicated concentrations of PX-12 for the indicated time points. (A) Figures indicate cell population in PX-12-treated HeLa cells at 72 h. (B) Cellular growth changes in HeLa cells as assessed by MTT assays. (C) Changes in the cell cycle distributions as assessed by DNA flow cytometric analysis at 72 h. ^*^P<0.05, compared with the control group. MTT, 3-(4,5-dimethylthiazol-2-yl)-2,5-diphenyltetrazolium bromide.

We first examined the effect of PX-12 on the growth of HeLa cells. After exposure to 1–10 μM PX-12 for 72 h, the population of HeLa cells was not affected at 1 μM PX-12, whereas the population of these cells was markedly decreased at 5–10 μM PX-12 (Fig. 1A). In addition, 5 and 10 μM PX-12 treatment induced cell death in HeLa cells (Fig. 1A). Based on MTT assays, the tested doses (1–30 μM) of PX-12 did not affect changes in cell growth at 24 h, whereas a high dose of 30 μM PX-12 significantly decreased the growth of HeLa cell at 48 h (Fig. 1B). At 72 h, 5–30 μM PX-12 significantly inhibited the growth of HeLa cells with an IC_50_ value (the half maximal inhibitory concentration) of ~7 μM at 72 h (Fig. 1B). When the cell cycle distributions were examined in PX-12-treated HeLa cells, 5 and 10 μM PX-12 significantly induced a G2/M phase arrest of the cell cycle at 72 h (Fig. 1C).

### Effects of PX-12 on cell death and MMP in HeLa cells

**Figure 2:**
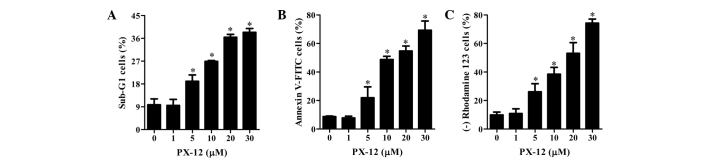
Effects of PX-12 on cell death and MMP in HeLa cells. Exponentially growing cells were treated with the indicated concentrations of PX-12 for 72 h. Percentages of (A) sub-G1 cells and (B) annexin V-positive cells as measured by FACStar flow cytometry. (C) The percentage of rhodamine 123-negative (loss of MMP) cells as measured by FACStar flow cytometry. ^*^P<0.05, compared with the control group. MMP, mitochondrial membrane potential; FITC, fluorescein isothiocyanate.

As shown in Fig. 2A, PX-12 increased the percentages of sub-G1 cells in a dose-dependent manner at 72 h. Treatment with 5–30 μM PX-12 increased the number of annexin V-FITC-positive cells, whereas 1 μM PX-12 did not increase the percentage of annexin V-FITC-positive cells (Fig. 2B). Cell death is closely associated with the collapse of MMP (29). As expected, the loss of MMP was observed in PX-12-treated HeLa cells (Fig. 2C). This result indicates that PX-12 damaged the membrane of mitochondria in HeLa cells.

### Effects of PX-12 on ROS and GSH levels in HeLa cells

**Figure 3:**
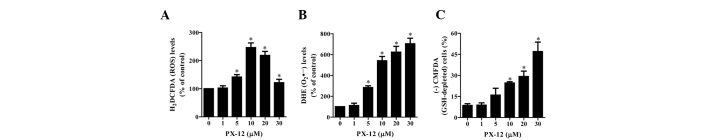
Effects of PX-12 on the intracellular ROS and GSH levels in HeLa cells. Exponentially growing cells were treated with the indicated concentrations of PX-12 for 72 h. ROS and GSH levels in HeLa cells were measured using a FACStar flow cytometer. (A) H_2_DCFDA (ROS) levels and (B) DHE (O_2_^•−^) levels as a percentage of the control. (C) The percentage of CMFDA-negative (GSH-depleted) cells. ^*^P<0.05, compared with the control group. ROS, reactive oxygen species; GSH, glutathione; H_2_DCFDA, 2′,7′-dichlorodihydrofluorescein diacetate; DHE, dihydroethidium; CMFDA, 5-chloromethylfluorescein diacetate.

To assess the intracellular ROS levels in PX-12-treated HeLa cells, we used H_2_DCFDA and DHE dyes. As shown in Fig. 3A, PX-12 significantly increased the intracellular ROS (H_2_DCFDA) levels in HeLa cells at 72 h. Among the tested concentrations, 10 μM PX-12 led to the maximum level of ROS (H_2_DCFDA) (Fig. 3A). Moreover, red fluorescence derived from DHE reflecting the intracellular O_2_^•−^ levels was markedly increased in PX-12-treated HeLa cells at 72 h (Fig. 3B). When intracellular GSH levels were measured in PX-12-treated HeLa cells using a CMFDA dye, 10–30 μM PX-12 significantly increased the number of GSH-depleted cells at 72 h; however, 5 μM PX-12 marginally induced GSH depletion (Fig. 3C).

### Effects of caspase inhibitors on cell death, MMP, O_2_^•−^ and GSH levels in PX-12-treated HeLa cells

**Figure 4:**
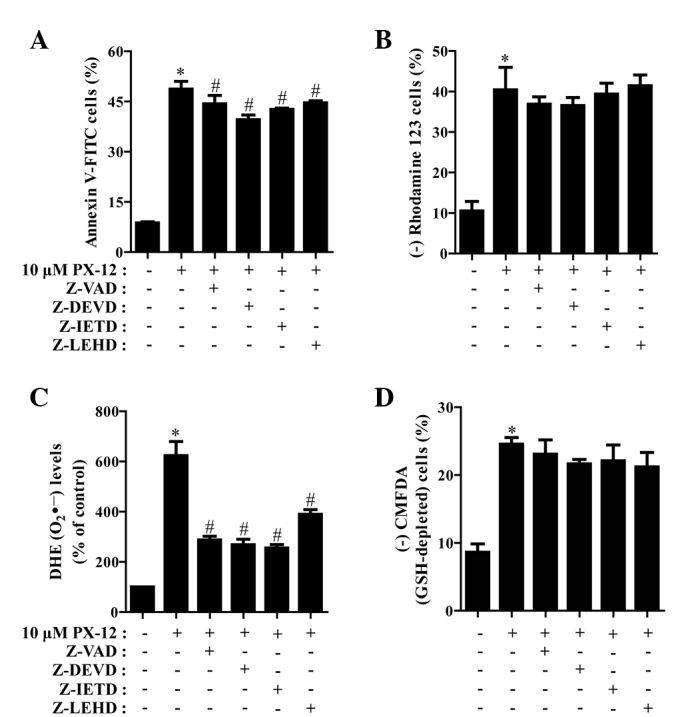
Effects of caspase inhibitors on cell death, MMP, O_2_^•−^ and GSH levels in PX-12-treated HeLa cells. Exponentially growing cells were treated with 10 μM PX-12 for 72 h following 1 h pre-incubation with 15 μM each caspase inhibitor. The number of annexin V/PI-stained cells, as well as MMP, O_2_^•−^ and GSH levels in HeLa cells were measured using a FACStar flow cytometer. Percentage of (A) annexin V-positive cells, (B) rhodamine 123-negative (loss of MMP) cells, (C) O_2_^•−^ (DHE) levels and (D) CMFDA-negative (GSH-depleted) cells. ^*^P<0.05, compared with the control group; ^#^P<0.05, compared with cells treated with PX-12 only. MMP, mitochondrial membrane potential; GSH, glutathione; PI, propidium iodide; DHE, dihydroethidium; CMFDA, 5-chloromethylfluorescein diacetate.

We determined which caspases were involved in HeLa cell death caused by PX-12. For this experiment, we selected 10 μM PX-12 as a suitable dose to differentiate the levels of cell death in the presence or absence of each caspase inhibitor. Based on a previous study (21), HeLa cells were pretreated with 15 μM caspase inhibitor for 1 h prior to treatment with PX-12. This dose did not significantly affect cell death in the control HeLa cells (data not shown). Treatment with all the tested caspase inhibitors (Z-VAD for pan-caspases, Z-DEVD for caspase-3, Z-IETD for caspase-8 and Z-LEHD for caspase-9) demonstrated the significant rescue of HeLa cells from PX-12-induced apoptosis at 72 h, as measured by the population of annexin V-FITC-positive cells (Fig. 4A). In addition, all the caspase inhibitors marginally, but not significantly, prevented the loss of MMP caused by PX-12 (Fig. 4B).

It was also investigated whether the levels of intracellular O_2_^•−^ and GSH in PX-12-treated HeLa cells were affected by treatment with each caspase inhibitor. As shown in Fig. 4C, all the caspase inhibitors significantly decreased O_2_^•−^ levels in PX-12-treated HeLa cells. Moreover, these caspase inhibitors marginally prevented GSH depletion in these cells (Fig. 4D).

### Effects of NAC and BSO on cell death, MMP, O_2_^•−^ and GSH levels in PX-12-treated HeLa cells

**Figure 5:**
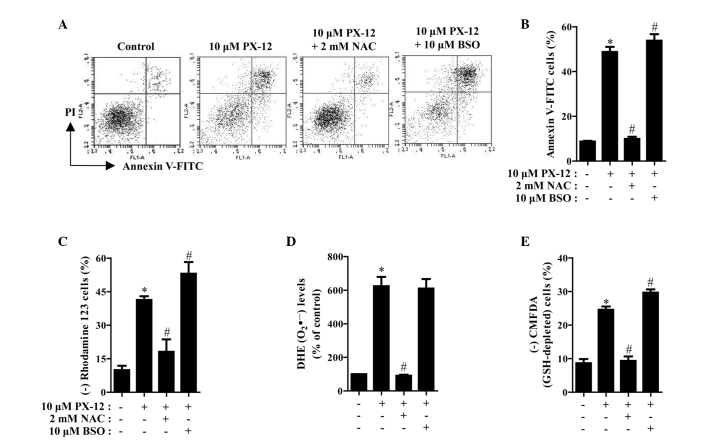
Effects of NAC and BSO on cell death, MMP, O_2_^•−^ and GSH levels in PX-12-treated HeLa cells. Exponentially growing cells were treated with 10 μM PX-12 for 72 h following 1 h pre-incubation with 2 mM NAC or 10 μM BSO. The number of annexin V/PI-stained cells, as well as MMP, O_2_^•−^ and GSH levels in HeLa cells were measured using a FACStar flow cytometer. (A) Each figure shows a representative for annexin V and/or PI stained cells. (B) The graph shows the percentage of annexin V-positive cells from A. (C) The graph shows the percentage of rhodamine 123-negative (loss of MMP) cells. (D and E) The graphs indicate O_2_^•−^ (DHE) levels as a percentage of the control (D) and the percentage of CMFDA-negative (GSH-depleted) cells (E). ^*^P<0.05, compared with the control group; ^#^P<0.05, compared with cells treated with PX-12 only. NAC, N-acetyl cysteine; BSO, L-buthionine sulfoximine; MMP, mitochondrial membrane potential; GSH, glutathione; PI, propidium iodide; DHE, dihydroethidium; CMFDA, 5-chloromethylfluorescein diacetate.

The effects of NAC or BSO on cell death and MMP in 10 μM PX-12-treated HeLa cells were assessed at 72 h. As shown in Fig. 5A and B, NAC significantly decreased the number of annexin V-FITC-positive cells in the PX-12-treated HeLa cell population, whereas BSO increased the number of these cells. NAC and BSO did not significantly affect cell growth and cell death in the control HeLa cells (data not shown). With respect to MMP, NAC significantly attenuated the loss of MMP caused by PX-12 whereas BSO enhanced, to a certain extent, the loss in these cells (Fig. 5C). Furthermore, it was determined whether the levels of intracellular O_2_^•−^ and GSH in PX-12-treated HeLa cells were affected by treatment with NAC or BSO. While NAC markedly decreased the level of O_2_^•−^ in PX-12-treated HeLa cells, BSO had no effect on the level of O_2_^•−^ in these cells (Fig. 5D). With regard to GSH levels, NAC markedly prevented GSH depletion caused by PX-12, whereas BSO intensified GSH depletion in these cells (Fig. 5E).

## Discussion

The aim of the present study was to assess the effects of PX-12 on cell growth and death in HeLa cells in association with ROS and GSH levels. Following exposure to PX-12 for 72 h, the IC_50_ value in HeLa cells was ~7 μM based on MTT assays. However, the tested doses of PX-12 did not show the growth inhibition of HeLa cells at 24 h and this effect was mild at 48 h. Therefore, the susceptibility of HeLa cells to PX-12 appeared to significantly increase after the incubation time of 48 h. DNA flow cytometric analysis indicated that 5 and 10 μM significantly induced a G2/M phase arrest of the cell cycle. As 20 and 30 μM PX-12 completely decreased cell growth, it was not possible to perform cell cycle analysis in HeLa cells. Similarly, PX-12 induced a G2/M phase arrest in B-cell lymphoma and breast cancer cells (7,30). We also observed that PX-12 induced a G2/M phase arrest in A549 and Calu-6 lung cancer cells (unpublished data). Therefore, the G2/M phase arrest in PX-12-treated cells was an underlying mechanism to suppress the growth of cancer cells, including HeLa cells.

PX-12 also increased the number of dead cells and annexin V-FITC-positive cells at 72 h, suggesting that PX-12-induced HeLa cell death occurred via apoptosis. Apoptosis is closely associated with the collapse of MMP (31). Our results demonstrated that PX-12 triggered the loss of MMP in HeLa cells in a dose-dependent manner. Furthermore, treatment with the caspase inhibitors investigated in this experiment significantly prevented HeLa cell death caused by PX-12. In particular, the caspase-8 inhibitor attenuated HeLa cell death. These data suggest that the mitochondrial pathway and cell death receptor pathway are together necessary for the complete induction of apoptosis in PX-12-treated HeLa cells. However, all the caspase inhibitors marginally, but not significantly prevented the loss of MMP caused by PX-12. These results implied that the loss of MMP by PX-12 may not be enough to fully induce apoptosis in HeLa cells under the inhibition of caspases by their inhibitors.

PX-12, as an inhibitor of Trx-1, increases ROS levels. It has been reported that PX-12 induces oxidative stress (32). Similarly, in the present study, the intracellular ROS levels, particularly those of O_2_^•−^, were significantly increased in PX-12-treated HeLa cells at 72 h. All caspase inhibitors demonstrating anti-apoptotic effects decreased the level of O_2_^•−^. These data indicated that the level of O_2_^•−^, among other ROS, is closely associated with apoptosis in PX-12-treated HeLa cells. Furthermore, NAC markedly prevented apoptotic cell death and the loss of MMP in PX-12-treated HeLa cells, accompanied by strongly decreasing O_2_^•−^ levels in these cells. Overall, these results suggest that PX-12-induced cell death is mediated by oxidative stress. GSH is an important intracellular antioxidant that protects cells from damage caused by free radicals, peroxides and toxins. It is able to remove O_2_^•−^ and provide electrons for glutathione peroxidase to reduce H_2_O_2_ to H_2_O. Apoptotic effects are inversely comparative to GSH content (33–35). In the current study, PX-12 increased the percentages of GSH-depleted cells at 72 h. NAC markedly prevented the depletion of GSH in PX-12-treated HeLa cells. Furthermore, BSO, which augmented apoptotic cell death and the loss of MMP in PX-12-treated HeLa cells, increased GSH depletion in these cells. These results support the hypothesis that the intracellular GSH content has a decisive effect on cell death (26,28,34). However, in the present study, caspase inhibitors marginally prevented GSH depletion in PX-12-treated HeLa cells. Therefore, the loss of GSH content appeared to be necessary, but not sufficient to fully induce apoptosis in PX-12-treated HeLa cells.

In conclusion, to the best of our knowledge, this is the first study to demonstrate that PX-12 inhibits the growth of HeLa cells via G2/M phase arrest, as well as apoptosis. This toxicological effect was associated with intracellular increases in ROS levels and GSH depletion. The present study provides an important insight into the toxicological effects of PX-12 on HeLa cells with respect to ROS and GSH levels.

## Abbreviations

ROS: reactive oxygen species
Trx: thiore doxin
GSH: glutathione
Z-VAD-FMK: benzyloxycarbonyl-Val-AlaAsp-fluoromethylketone
Z-DEVD-FMK: benzyloxycarbonyl- Asp-Glu-Val-Asp-fluoromethylketone
Z-IETD-FMK: benzyl oxycarbonyl-Ile-Glu-Thr-Asp-fluoromethylketone
Z-LEHD-FMK: benzyloxycarbonyl-Leu-Glu-His-Asp-fluoromethylketone
NAC: N-acetyl cysteine
BSO: L-buthionine sulfoximine
MMP: mitochondrial membrane potential
MTT: 3-(4,5-dimethylthiazol2-yl)-2,5-diphenyltetrazolium bromide
FITC: fluorescein isothiocyanate
PI: propidium iodide
H_2_DCFDA: 2′,7′-dichlorodihydrofluorescein diacetate
DHE: dihydroethidium
CMFDA: 5-chloromethylfluorescein diacetate
